# Fxr1 Deletion from Cortical Parvalbumin Interneurons Modifies Their Excitatory Synaptic Responses

**DOI:** 10.1523/ENEURO.0363-24.2024

**Published:** 2025-01-10

**Authors:** Katherine S. Scheuer, Anna M. Jansson, Minjie Shen, Xinyu Zhao, Meyer B. Jackson

**Affiliations:** ^1^Department of Neuroscience, University of Wisconsin-Madison, Madison, Wisconsin 53705; ^2^Waisman Center, University of Wisconsin-Madison, Madison, Wisconsin 53705; ^3^The State Key Laboratory of Medical Neurobiology, MOE Frontiers Center for Brain Science, and the Institutes of Brain Science, Fudan University, Shanghai 200032, China

**Keywords:** barrel cortex, FXR1, genetically encoded voltage indicator, hVOS, parvalbumin interneurons, somatosensory cortex

## Abstract

Fragile X autosomal homolog 1 (FXR1), a member of the fragile X messenger riboprotein 1 family, has been linked to psychiatric disorders including autism and schizophrenia. Parvalbumin (PV) interneurons play critical roles in cortical processing and have been implicated in FXR1-linked mental illnesses. Targeted deletion of FXR1 from PV interneurons in mice has been shown to alter cortical excitability and elicit schizophrenia-like behavior. This indicates that FXR1 regulates behaviorally relevant electrophysiological functions in PV interneurons. We therefore expressed a genetically encoded hybrid voltage sensor in PV interneurons and used voltage imaging in slices of mouse somatosensory cortex to assess the impact of targeted FXR1 deletion. These experiments showed that PV interneurons lacking FXR1 had excitatory synaptic potentials with larger amplitudes and shorter latencies compared with wild type. Synaptic potential rise-times, decay-times, and half-widths were also impacted to degrees that varied between cortical layer and synaptic input. Thus, FXR1 modulates the responsiveness of PV interneurons to excitatory synaptic inputs. This will enable FXR1 to control cortical processing in subtle ways, with the potential to influence behavior and contribute to psychiatric dysfunction.

## Significance Statement

Parvalbumin interneurons have been implicated in schizophrenia and autism. The RNA-binding protein FXR1, a member of the fragile X protein family, has been linked to mental illnesses and disabilities. Voltage imaging from parvalbumin interneurons in cortical slices revealed that targeted ablation of FXR1 from these neurons alters the amplitude and dynamics of their excitatory synaptic responses. These changes have the potential to alter circuit processing and behavior and may be relevant to FXR1-linked mental illnesses.

## Introduction

Fragile X autosomal homolog 1 (*FXR1*) encodes an RNA-binding protein in the same family as fragile X messenger riboprotein 1 (*FMR1*), the gene responsible for fragile X syndrome (FXS; Siomi et al., 1995; Hoogeveen and Oostra, 1997). FXR1 both regulates and is regulated by microRNAs (Cheever et al., 2010; X. L. Xu et al., 2011) and can activate as well as repress translation (Garnon et al., 2005; Vasudevan et al., 2007). It has been linked to a wide range of disorders impacting human health including muscular diseases (Davidovic et al., 2008; Mroczek et al., 2022; Mueller et al., 2023) and many types of cancer (Comtesse et al., 2007; Qian et al., 2015; Jiang et al., 2016; Q. Li et al., 2016; Cao et al., 2019; Huang et al., 2023). Although not altered in individuals with FXS (Siomi et al., 1995), FXR1 has been implicated in neurodevelopmental and psychiatric disorders (Mendez-Albelo et al., 2024). Genome-wide association studies identified *FXR1* as a risk variant for schizophrenia (Schizophrenia Working Group of the Psychiatric Genomics, 2014), and *FXR1* mRNA levels are reduced in postmortem brain in some individuals with schizophrenia and bipolar disorders (Shen et al., 2021). An interaction between polymorphisms in *FXR1* and one of its regulators, glycogen synthase kinase 3β, predicts symptom severity in schizophrenia and bipolar disorder (Benedict et al., 2017; Rampino et al., 2021). FXR1 has been shown to be involved in RNA editing of autism-related genes (Tran et al., 2019). The links between FXR1 and psychiatric disorders provide a strong incentive to gain an understanding of its roles in neural function.

Studies of FXR1 have been hindered by the perinatal lethality of its knock-out (KO; Mientjes et al., 2004). Changes in FXR1 expression alter neuronal morphology, receptor expression, action potential firing, plasticity, and behavior (Cook et al., 2014; Khlghatyan et al., 2018b, 2020; Shen et al., 2021). However, effects can vary based on cell type and brain region. For example, spatial memory improves with FXR1 conditional KO (cKO) in excitatory neurons (Cook et al., 2014) but worsens with FXR1 cKO in a class of inhibitory interneurons defined by expression of the Ca^2+^-binding protein parvalbumin (PV; Shen et al., 2021). Furthermore, PV interneuron-specific FXR1 cKO alters gamma oscillations in the prefrontal cortex and hippocampus (but not in entorhinal cortex) and impacts cellular excitability and expression of the T-type calcium channel Ca_v_3.2 (Shen et al., 2021). Because PV interneurons are fast-spiking, they are thought to control circuit timing functions such as temporal integration (Gonzalez-Burgos et al., 2015). PV interneurons in the somatosensory cortex are reduced in numbers (C. Z. Wang et al., 2008; Deemyad et al., 2022) and exhibit impaired GABA release (Cellot and Cherubini, 2014) in mouse models of autism spectrum disorder and schizophrenia. These neurons also regulate excitation/inhibition balance (Hu et al., 2014; Ferguson and Gao, 2018) and gamma oscillations (Tallon-Baudry et al., 1998; Cardin et al., 2009; McNally and McCarley, 2016; Cardin, 2018). There is strong evidence for both gamma oscillation and PV interneuron dysfunction in FXR1-associated disorders including autism spectrum disorder and schizophrenia (Gonzalez-Burgos et al., 2015; Ferguson and Gao, 2018; Lauber et al., 2018; Nahar et al., 2021).

Despite the potential links of both FXR1 and PV interneurons to psychiatric disorders, to our knowledge there has been only one prior study of the role of FXR1 specifically in PV interneurons (Shen et al., 2021). We therefore investigated the impact of FXR1 on the responses of PV interneurons to excitatory synaptic inputs in mouse primary somatosensory cortex. PV interneuron morphology, circuitry, and function vary between cortical layers (X. Xu and Callaway, 2009; P. Li and Huntsman, 2014; Naka and Adesnik, 2016; Staiger and Petersen, 2021; Canales et al., 2023; Scheuer et al., 2024). Thus, it is important to test the impact of molecular deficits in different layers. To address these issues, we used the genetically encoded hybrid voltage sensor (hVOS) to compare wild-type (WT) and FXR1 cKO PV interneurons in the barrel cortex (BC), a region of the somatosensory cortex with well-established roles in processing whisker sensory inputs. hVOS imaging has subcellular spatial resolution and submillisecond temporal resolution (Chanda et al., 2005; Bradley et al., 2009; D. Wang et al., 2010). Simultaneous patch-clamp recording has shown that hVOS reports voltage changes and tracks action potentials with high temporal fidelity (D. Wang et al., 2010; Ghitani et al., 2015; Bayguinov et al., 2017). hVOS imaging reveals electrically evoked excitatory postsynaptic potentials (EPSPs) in multiple cells simultaneously across cortical layers (Bayguinov et al., 2017; Canales et al., 2023; Scheuer et al., 2023). We found that selective FXR1 ablation from PV interneurons increased the amplitude and decreased the latency of their EPSPs. Rise-time, decay-time, and half-width were also altered selectively in PV interneurons residing in different cortical layers and in response to excitation from different layers. These results reveal distinct layer- and input-specific roles for FXR1 in controlling the responses of PV interneurons to excitatory synaptic inputs.

## Materials and Methods

### Ethical approval

All animal procedures were approved by the Institutional Animal Care Committee.

### Animals

PV Cre driver mice (B6.129P2-*Pvalb^tm1(cre)Arbr^*/J; https://www.jax.org/strain/017320) were bred with Ai35-hVOS1.5 Cre reporter mice [C57BL/6-*Gt(ROSA)26Sor^tm1(CAG-hVOS1.5)Mbja^*/J; https://www.jax.org/strain/031102; Bayguinov et al., 2017] and FXR1 floxed mice (Mientjes et al., 2004; Shen et al., 2021) to create transgenic animals with PV interneuron-specific FXR1 deletion and hVOS probe expression in the same cells. Because in these animals only PV interneurons expressed Cre recombinase, the FXR1-encoding sequence and the STOP codon before the hVOS-encoding sequence were both excised specifically in these cells. Experiments were conducted on WT and cKO mice over the same time period, with all animals drawn from the same colony (except one WT). WT and cKO mice were matched based on birthdate and sex. A parallel set of experiments with floxed FXR1 and Ai14 Cre reporter mice (JAX 007914) demonstrated that 92% and 81% of fluorescent protein expressing cells were PV positive in control and cKO, respectively, and that FXR1 deletion did not reduce the number of PV positive cells (Shen et al., 2021).

### Hybrid voltage sensor

The hVOS probe is cerulean fluorescent protein (CeFP) tethered to the inner leaflet of the plasma membrane with a truncated h-ras motif (D. Wang et al., 2010). Dipicrylamine (DPA), a small hydrophobic anion which partitions into cell membranes, is present in the recording solution. Depolarization moves DPA within the membrane, and in cells expressing hVOS probe, DPA is pushed toward the CeFP to reduce fluorescence as a result of Förster resonance energy transfer. Repolarization moves DPA back and away from the CeFP allowing fluorescence to increase (Chanda et al., 2005; D. Wang et al., 2010). Changes in fluorescence thus report voltage changes. Ai35-hVOS1.5 Cre reporter animals express hVOS probe in targeted cells, including PV interneurons, with high specificity and selectivity (Bayguinov et al., 2017). hVOS records unitary synaptic potentials (Ma et al., 2021; Canales et al., 2023), and a submillisecond response time enables the tracking of action potentials with high temporal fidelity (Chanda et al., 2005; Bradley et al., 2009; Ghitani et al., 2015; Ma et al., 2019).

### Slice preparation

Six- to ten-week-old male or female mice were deeply anesthetized with isoflurane and killed by cervical dislocation. Brains were rapidly dissected and placed into ice-cold cutting solution (in mM: 10 glucose, 125 NaCl, 4 KCl, 1.25 NaH_2_PO_4_, 26 NaHCO_3_, 6 MgSO_4_, 1 CaCl_2_) bubbled with 95% O_2_/5% CO_2_. Coronal slices 300 μm thick containing BC were cut in ice-cold cutting solution using a Leica VT1200S Vibratome. Slices recovered for at least an hour at room temperature in 95% O_2_/5% CO_2_ bubbled artificial cerebrospinal fluid (ACSF), which has the same composition as cutting solution but with 1.3 mM MgSO_4_, 2.5 mM CaCl_2_, and 4 μM DPA.

### Voltage imaging

Slices were viewed with a BX51 Olympus microscope while continuously perfusing with 95% O_2_/5% CO_2_ bubbled ACSF. Excitation light from an LED (Prizmatix) with peak emission at 435 nm was passed through a CeFP filter cube. To reduce baseline fluorescence as needed (due to camera saturation), additional 435 nm filters with 5 and/or 10 nm bandpass were inserted. A Kiralux CMOS camera (Thorlabs) captured high-resolution gradient-contrast and fluorescence images. Fluorescence changes were recorded with a CCD-SMQ camera (RedShirt Imaging/SciMeasure) with 80 × 80 spatial resolution. Images were acquired and analyzed with custom software (Chang, 2006) at 2,000 frames per second. All displayed records were five-trial averages acquired at 15 s intervals. Slices were stimulated extracellularly with 180 μs pulses from a stimulus isolator (World Precision Instruments) through fire-polished KG-33 glass electrodes (King Precision Glass) with ∼6–8 μm diameter tips. A pulse amplitude of 200 µA was selected as this generally produced strong responses without causing damage (Scheuer et al., 2023). Stimulating electrodes were filled with ACSF and positioned in L2/3 or L4 with a micromanipulator.

### Data processing and analysis

Fluorescence intensity was divided by resting light intensity to determine Δ*F*/*F* and processed digitally using a spatial filter with *σ* = 1 and a nine-point binomial temporal filter. The dense arborization of PV interneurons (Fukuda and Kosaka, 2003) makes their cell bodies difficult to locate in resting fluorescence images, so responsive PV interneurons were identified based on combined analysis of maps of both peak response amplitude and signal-to-noise ratio (SNR; Scheuer et al., 2024). SNR was calculated in fluorescence versus time traces for each pixel by dividing the maximum stimulus-evoked change in fluorescence by the prestimulus root-mean-square baseline noise. Responsive PV interneurons were identified as small contiguous groups of pixels with both amplitude and SNR greater than predetermined cutoffs and with sizes smaller than PV interneuron somata (3 pixels across, 19 μm; Y. Wang et al., 2002; Selby et al., 2007; Kooijmans et al., 2020). Mean pixel counts in these groups were 2.71 ± 0.96 for WT and 2.83 ± 1.10 for cKO (mean ± SD), giving mean areas of 108 and 113 μm^2^, respectively. Thus, the area of a region determined by this process is a bit less than the area of a PV interneuron soma, and the smaller size reflects the conservative criteria for only including pixels within the same SNR level (as determined by *k*-means cluster analysis described below); pixels with lower SNR that may be at a cell's edge were thus not included. All groups of pixels were separate; groups that bordered one another, even at a single vertex, were excluded to avoid mixing signals from multiple neurons. One-dimensional *k*-means clustering was then performed on pixel SNR, and all pixels falling within *k*-means defined clusters with average SNR below a predetermined cutoff of 5 were excluded. This automated procedure in which we applied spatial geometric constraints together with statistical clustering of responses allowed us to identify small contiguous group of pixels likely to arise from individual responsive PV interneurons. Signals from a group of pixels selected in this way were averaged to produce traces of fluorescence versus time. A full account of this method can be found in Scheuer et al. (2024), along with validation of single neuron detection by showing that response amplitude and half-width are independent of distance from the site of stimulation. When regions seen with voltage imaging contain multiple neurons, synchrony declines with distance, leading to a reduction in amplitude and broadening of half-width (Ma et al., 2017; Scheuer et al., 2023). PV interneurons obscured by the stimulating electrode were excluded. Neurons within 45 μm of the stimulating electrode or with latency <1 ms are likely to result from direct stimulation, so they were also excluded. We discarded seven neurons because noise interfered with waveform analysis and parameter calculation, and 17 neurons because their response amplitudes were more than three standard deviations above the mean. These neurons were in particularly dark areas near the edge of the field of view where dim light resulted in high noise, and dividing by resting light produced artificially large amplitudes. In summary, the final yield was 2,200 (1,084 WT, 1,116 cKO) regions of interest that were accepted as responsive neurons suitable for analysis with an SNR ranging from 5.06 to 26.25. These regions of interest had an average area of 113 μm^2^, and regions with a linear dimension >19 μm (or area > 360 μm^2^) were excluded from analysis as they are likely to contain more than one neuron. We cannot rule out the possibility that some of these small and separate regions contain another overlapping soma or comprise converging processes from multiple neurons.

**Figure 1.**
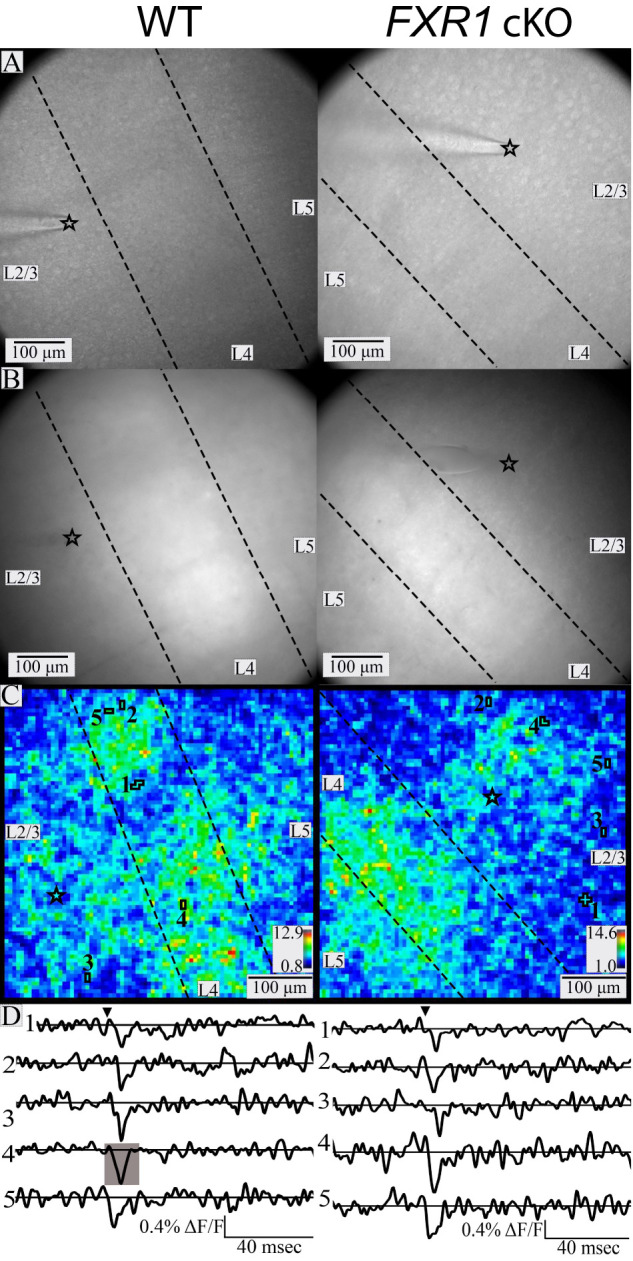
PV interneuron responses in coronal BC slices from a WT mouse (left) and a FXR1 cKO mouse (right). Gradient-contrast images (***A***) and fluorescence images (***B***) show L2/3 through L5. ***C***, SNR heatmaps from experiments with the same slices shown in ***A*** and ***B***. Warmer colors (yellow-orange-red) correspond to higher SNR (scale at bottom right). In each slice, black outlines and numbers indicate five selected responsive PV interneurons. In ***A–C***, black stars indicate the tip of the stimulating electrode (visible in ***A***, faint in ***B***), and dashed lines mark layer boundaries. ***D***, Traces of fluorescence versus time for the five WT and five FXR1 cKO PV interneurons outlined in ***C***. Following stimulation (triangle at top), all cells show clear decreases in fluorescence corresponding to depolarization. The shaded trace was used in Figure 2 to illustrate determination of EPSP parameters.

Boundaries between cortical layers were visually identified in gradient-contrast (Fig. 1*A*) and fluorescence (Fig. 1*B*) images based on cell density and the presence of barrels in L4 (Woolsey and Van der Loos, 1970; Feldmeyer, 2012). For both WT (Fig. 1, left) and cKO (Fig. 1, right), stimulation in L2/3 or L4 elicited responses across L2/3 through L5, as illustrated in maps of SNR encoded as color (Fig. 1*C*). SNR maps generally reveal responsive neurons more clearly than amplitude maps. Five selected responsive cells identified by the process described above are highlighted with black outlines in both maps. Some of the brightest regions were too large to be a single cell and so we did not use them, despite the fact that they produced strong signals with high SNR. The selected cells are also indicated by numbers, and traces of fluorescence versus time from these cells (Fig. 1*D*) indicate that PV interneurons identified as described above display decreases in fluorescence following stimulation (time marked with a triangle), as depolarization moves DPA closer to the fluorescent protein of the hVOS probe and quenches fluorescence emission. These responses were judged to be EPSPs based on their amplitude and kinetics as well as their previously demonstrated sensitivity to glutamate receptor blockade (Canales et al., 2023).

**Figure 2.**
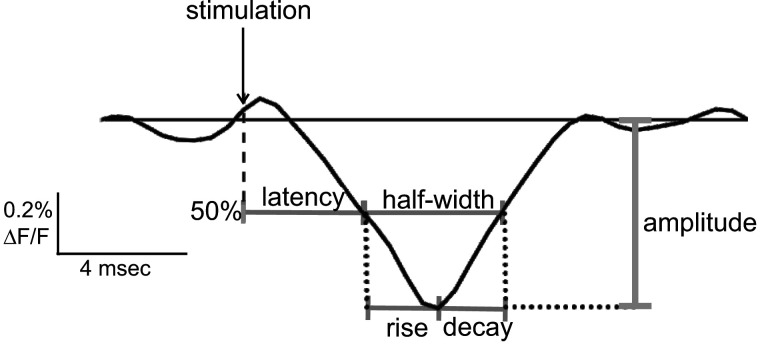
An EPSP with parameters illustrated in an expanded view of the gray boxed trace in Figure 1*D* (cell #4, WT). Amplitude is the maximum change in fluorescence from baseline, latency is the time from stimulation to half-maximal change in fluorescence, rise-time is the time from half-maximal change during depolarization to peak; decay-time is the time from peak to half-maximal change during repolarization; and half-width is the time between the two half-maximal levels.

EPSP parameters were determined from traces of fluorescence versus time as illustrated in Figure 2. Amplitude was taken as the maximal stimulus-evoked change in fluorescence (Δ*F*/*F*), and latency was taken as the time from stimulation to half-maximal change during depolarization. Latency increases with distance from the site of stimulation due to conduction (Scheuer et al., 2023). We therefore divided latency by distance to obtain a distance-normalized latency (see Results). Rise-time was taken as the time from leading half-maximal level to peak, and decay-time was the time from peak to the following half-maximal change. Half-width was the time between half-maximal changes preceding and following the peak. Parameter values are all presented as mean ± SE.

### Experimental design and statistical tests

**Figure 3.**
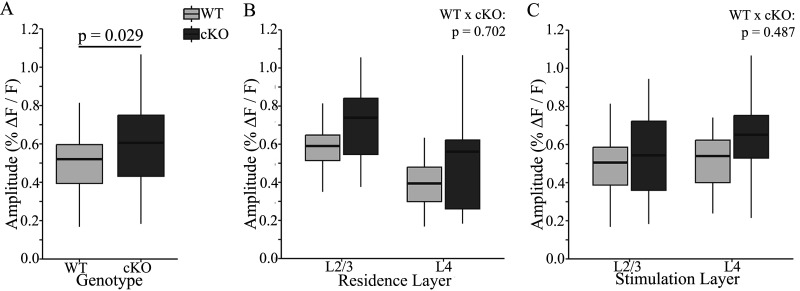
FXR1 cKO increases EPSP amplitudes of PV interneurons. ***A***, Pooled response amplitude from cKO slices (dark gray) is significantly larger than WT (light gray, *p* = 0.029). ***B***, Breaking down the results into layers where PV interneurons reside, amplitude is significantly larger for EPSPs of PV interneurons residing in L2/3 compared with L4 (*F*_(1,102)_ = 38.226, *p* < 0.001). There was no significant difference between layers in the impact of genotype (*p* = 0.702). ***C***, Stimulation layer does not significantly affect EPSP amplitude (*p* = 0.475), and there is no significant interaction between stimulation layer and genotype (*p* = 0.487).

**Figure 4.**
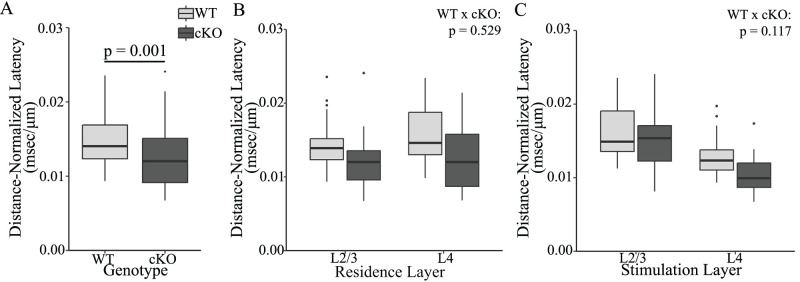
FXR1 cKO decreases distance-normalized latency of EPSPs in PV interneurons. ***A***, Pooled EPSPs from cKO slices (dark gray) have significantly shorter distance-normalized latencies than WT (light gray) (*p* = 0.001). ***B***, PV interneurons residing in L2/3 and L4 have indistinguishable distance-normalized latencies, and genotype has no effect (*p* = 0.529). ***C***, Distance-normalized latencies are shorter for responses to L4 stimulation compared with L2/3 stimulation in WT (*F*_(1,102)_ = 49.403, *p* < 0.001), and there was no significant difference between layers in the impact of genotype (*p* = 0.117). Here and in other figures, filled circles represent outlying points (see Materials and Methods).

**Figure 5.**
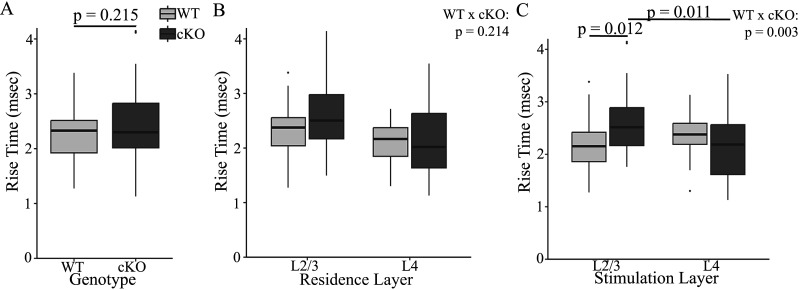
***A***, EPSP rise-times are indistinguishable between the pooled WT and FXR1 cKO data set (*p* = 0.215). ***B***, There is no significant difference between layers in the impact of genotype (*p* = 0.214). ***C***, Rise-times of PV interneuron EPSPs evoked by stimulation in L2/3 were longer in cKO slices compared with WT in L2/3 (*p* = 0.012) or L4 of cKO (*p* = 0.011). There was a significant difference between stimulation layers in the impact of genotype (*p* = 0.003).

**Figure 6.**
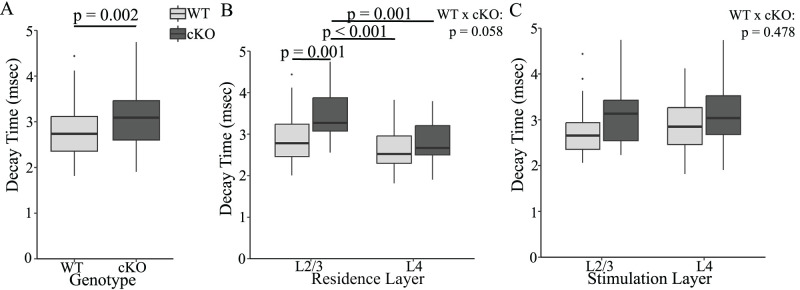
***A***, In pooled data FXR1 cKO increases PV interneuron EPSP decay-time (*p* = 0.002). ***B***, FXR1 cKO increases decay-time of PV interneurons residing in L2/3 but not in L4. FXR1 cKO PV interneurons have EPSPs with longer decay-times in L2/3 than in L4 (*p* = 0.001). These decay-times are also longer than both L2/3 (*p* = 0.001) and L4 (*p* < 0.001) of WT. The interaction between residence layer and genotype was near the significance threshold (*p* = 0.058). ***C***, Decay-time did not vary significantly based on stimulation layer (*F*_(1,101)_ = 0.115, *p* = 0.735), and there was no significant interaction between stimulation layer and genotype (*p* = 0.478).

**Figure 7.**
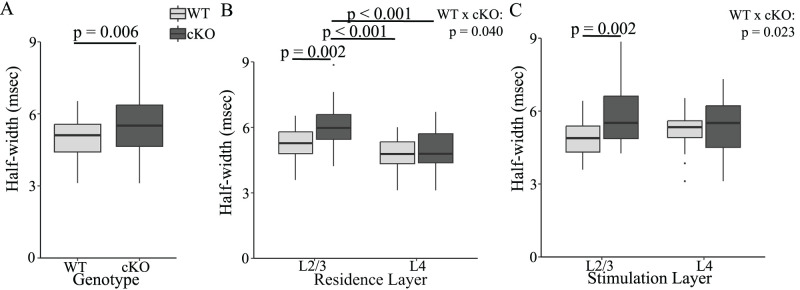
***A***, In pooled data FXR1 cKO increased EPSP half-width (*p* = 0.006). ***B***, In cKO slices half-widths in L2/3 were broader than in L4 (*p* < 0.001). The L2/3 cKO half-width was also broader than in both L2/3 WT (*p* = 0.002) and L4 (*p* < 0.001) from WT. There was a significant difference between residence layers in the impact of genotype (*p* = 0.040). ***C***, EPSPs elicited by L2/3 stimulation had longer half-widths in cKO slices compared with WT (*p* = 0.002) but half-widths of EPSPs elicited by L4 stimulation were indistinguishable. There was a significant difference between stimulation layers in the impact of genotype (*p* = 0.023).

Data were collected from 71 slices (38 WT, 33 cKO) from 14 animals (7 WT, 7 cKO; 43% female in both groups). For stimulation in L2/3 and L4, we recorded responses in both these layers and tested hypotheses that the parameters illustrated in Figure 2 varied based on residence layer and stimulation layer. For a given slice, parameters from PV interneurons in layers with eight or more responsive cells were averaged for each layer and that layer average was taken as the unit of analysis for statistical testing (*n* = 55 WT; *n* = 54 cKO). Parameter normality was evaluated with the Shapiro–Wilk tests. Amplitude (*W* = 0.985, *p* = 0.269), half-width (*W* = 0.986, *p* = 0.303), and rise-time (*W* = 0.979, *p* = 0.086) were normally distributed, and distance-normalized latency (*W* = 0.989, *p* = 0.504) and decay-time (*W* = 0.991, *p* = 0.676) were log-normally distributed. Data points >1.5-fold outside the interquartile range (outliers) were plotted separately as small filled circles in the box and whisker plots of Figures 3–7. Outlier points were included in the analysis.

Levene's tests were used to compare between-group variance. Variance did not differ significantly between sexes (amplitude: *F*_(1,107)_ = 1.742, *p* = 0.190; half-width: *F*_(1,107)_ = 0.065, *p* = 0.799; distance-normalized latency: *F*_(1,107)_ = 3.253, *p* = 0.074; rise-time: *F*_(1,107)_ = 0.484, *p* = 0.488; decay-time: *F*_(1,107)_ = 0.134, *p* = 0.715), so the effect of sex on parameters was evaluated with *t* tests. Sex had no significant effect on amplitude (*t*_(106.98)_ = 0.731, *p* = 0.466), half-width (*t*_(105.91)_ = −1.986, *p* = 0.050), distance-normalized latency (*t*_(106.68)_ = −1.055, *p* = 0.294), rise-time (*t*_(106.97)_ = −1.717, *p* = 0.089), or decay-time (*t*_(101.44)_ = −1.703, *p* = 0.092). Variance did not differ significantly between genotypes, PV interneuron residence layer, and stimulation layer for half-width (*F*_(7,101)_ = 1.237, *p* = 0.290), distance-normalized latency (*F*_(7,101)_ = 1.223, *p* = 0.297), rise-time (*F*_(7,101)_ = 1.297, *p* = 0.260), or decay-time (*F*_(7,101)_ = 0.181, *p* = 0.989), and the effect of these factors on response parameters was therefore evaluated with ANOVA and post hoc Tukey's honestly significant difference tests. Variance did differ significantly based on genotype, PV interneuron residence layer, and stimulation layer for amplitude (*F*_(7,101)_ = 2.323, *p* = 0.031). The effect of these variables on amplitude was therefore assessed with bootstrapped ANOVA tests using the R package pbANOVA (Alver and Zhang, 2023).

### Code accessibility

Data and code relevant to this study run on a standard PC under Windows and can be accessed from GitHub at https://github.com/ksscheuer/FXR1 and Zenodo at https://doi.org/10.5281/zenodo.14342804

## Results

Voltage imaging revealed stimulus-evoked EPSPs in PV interneurons spread over considerable distances throughout BC slices prepared from both WT and FXR1 cKO mice (Fig. 1*C*). We selected fields of view to maximize areas of L2/3 and L4 in order to acquire a large amount of data from these layers. L5 was also in view and we observed EPSPs there, but due to the smaller area in view, the numbers of neurons in L5 did not provide the statistical power needed for the present goals. Here we focused on neurons residing in L2/3 and L4. For each EPSP parameter (Fig. 2), we first compared pooled WT data against pooled FXR1 cKO data. We then separated the data to evaluate the impact of FXR1 cKO on PV interneurons residing in different layers and to evaluate its impact on responses to stimulation in different layers. We note that we recently published a study focusing on PV interneuron EPSP heterogeneity in WT (Scheuer et al., 2024). The present study used a subset of the data of that study from animals matched by date of birth and sex to our FXR1 cKO animals.

### FXR1 cKO increases amplitude and decreases latency

In pooled data, PV interneurons in FXR1 cKO slices have EPSPs with 20% larger amplitudes than EPSPs from WT slices (Fig. 3*A*; WT: Δ*F*/*F* = 0.499 ± 0.020%; cKO: Δ*F*/*F* = 0.597 ± 0.031%, *p* = 0.029). This difference between genotypes persisted when we separated data according to whether neurons reside in L2/3 or L4 and whether slices were stimulated in L2/3 or L4. In WT slices PV interneurons residing in L2/3 have EPSPs with larger amplitudes than those in L4 (Scheuer et al., 2023). Figure 3*B* illustrates this trend for our present data in both WT and FXR1 cKO; when the data from the two genotypes were combined, the amplitudes were larger in L2/3 than those in L4 (*p* < 0.001). Furthermore, genotype had no significant impact on the difference between residence layers (*p* = 0.702). Turning to stimulation layer, in both WT (Scheuer et al., 2023) and FXR1 cKO, the EPSPs evoked by stimulation in L2/3 and L4 had similar amplitudes. As with residence layer, there was no significant interaction between genotype and stimulation layer (Fig. 3*C*; *p* = 0.487). In summary, FXR1 cKO broadly increases EPSP amplitude in PV interneurons of BC.

Because of the importance of timing to PV interneuron function, we examined the impact of FXR1 on response latency. As mentioned in Materials and Methods, because latency includes axonal conduction time, it increases with distance from the stimulating electrode (Scheuer et al., 2023). Distance must therefore be taken into account. The average distance from responding neurons in L2/3 to the site of stimulation in L4 was 254 ± 5.2 μm, which is longer than the average distance of 183 ± 5.3 μm for responding neurons in L4 to the site of stimulation in the same layer. Although average distances were similar between WT (214 ± 6.5 μm/ms) and cKO (226 ± 6.3 μm/ms), the wide range of distances across a slice will make raw latencies too variable to discern meaningful differences. Latency and distance were strongly and significantly correlated (*F*_(1,107)_ = 54.7, *p* < 0.001), so for each PV interneuron we divided raw latency (Fig. 2) by distance to obtain a distance-normalized latency. In the pooled data, distance-normalized latency in WT slices (0.0150 ± 0.0005 ms/μm) was significantly longer than that in FXR1 cKO slices (0.0125 ± 0.0005 ms/μm; Fig. 4*A*; *p* = 0.001). In WT slices EPSPs in L2/3 and L4 have similar distance-normalized latencies (Scheuer et al., 2023), and we see this again in FXR1 cKO (Fig. 4*B*; *F*_(1,101)_ = 0.594, *p* = 0.443). There was no significant interaction between genotype and residence layer (Fig. 4*B*; *F*_(1,101)_ = 0.400, *p* = 0.529). In WT, distance-normalized latencies of EPSPs elicited by L2/3 stimulation were significantly longer than EPSPs elicited by L4 stimulation (Scheuer et al., 2023). This was also true when WT and FXR1 cKO data were pooled (*p* < 0.001). Again there was no significant interaction between genotype and stimulation layer (*F*_(1,101)_ = 2.498, *p* = 0.117). Thus, FXR1 cKO resulted in a global reduction in EPSP latency.

### FXR1 cKO increases rise-time, decay-time, and half-width in a layer-specific manner

Unlike amplitude and distance-normalized latency, FXR1 deletion impacted EPSP rise-time, decay-time, and half-width differently depending on residence and/or stimulation layer. We did not see a difference between rise-times of WT and cKO in pooled data (Fig. 5*A*; *F*_(1,101)_ = 1.560, *p* = 0.215). In WT mice, PV interneurons residing in L2/3 had been shown to have longer EPSP rise-times than in L4 (Scheuer et al., 2024), and this was seen again here when WT and FXR1 cKO data were pooled (Fig. 5*B*; *F*_(1,101)_ = 11.605, *p* = 0.001). This difference in rise-time between layers was indistinguishable between WT and FXR1 cKO (*F*_(1,101)_ = 1.567, *p* = 0.214). Turning to stimulation layer (Fig. 5*C*), we found that EPSPs elicited by L2/3 stimulation had longer rise-times in cKO slices (2.62 ± 0.136 ms) than those in WT slices (2.19 ± 0.078 ms, *p* = 0.012). In contrast, EPSP rise-times in WT were indistinguishable between stimulation layers (Scheuer et al., 2024), but in FXR1 cKO EPSP rise-times elicited by L4 stimulation (2.19 ± 0.124 ms) were significantly shorter than those with L2/3 stimulation (*p* = 0.011). The impact of genotype differed significantly between stimulation layers (Fig. 5*C*; *F*_(1,101)_ = 9.085, *p* = 0.003). Thus, the principal consequence of FXR1 cKO was a prolongation EPSP rise-times of responses to L2/3 stimulation. Deletion of FXR1 affected rise-times differently between layers, resulting in a difference between rise-times elicited by stimulation of different layers. Such a difference was not evident in WT EPSP rise-times.

In the pooled PV interneuron data, EPSP decay-times were significantly longer in FXR1 cKO than those in WT (Fig. 6*A*; WT: 2.79 ± 0.076; cKO: 3.14 ± 0.088; *F*_(1,101)_ = 10.349, *p* = 0.002), and the breakdown pointed to a dependence on residence layer rather than stimulation layer. Consistent with our previous report (Scheuer et al., 2024), WT EPSP decay-times were similar between residence layers (Fig. 6*B*; *p* = 0.621) and stimulation layers (Fig. 6*C*; *F*_(1,101)_ = 0.115, *p* = 0.735). In contrast, in FXR1 cKO the decay-times in L2/3 (3.45 ± 0.119 ms) were significantly longer than those in L4 (Fig. 6*B*; 2.82 ± 0.098 ms, *p* = 0.001). The decay-times in L2/3 of FXR1 cKO were also longer than the decay-times in both layers of WT (L2/3, 2.87 ± 0.106 ms, *p* = 0.001; L4, 2.69 ± 0.107 ms, *p* < 0.001). The interaction between genotype and PV interneuron residence layer was weak and slightly above the threshold for significance (Fig. 6*B*; *F*_(1,101)_ = 3.673, *p* = 0.058). Stimulating different layers in WT elicited EPSPs with indistinguishable decay-times (Scheuer et al., 2023), and this was evident again in WT and FXR1 cKO (Fig. 6*C*). There was no interaction between stimulation layer and genotype (*p* = 0.478). Thus, as noted above for rise-times, eliminating FXR1 affected PV interneuron decay-times differently in different layers. FXR1 cKO increased EPSP decay-times in PV interneurons residing in L2/3 but not in L4 (Fig. 6*B*).

Finally, EPSP half-width was longer in cKO slices than in WT slices (Fig. 7*A*; *F*_(1,101)_ = 7.889, *p* = 0.006), and the impact of FXR1 deletion differed significantly between residence layers (Fig. 7*B*; *F*_(1,101)_ = 4.344, *p* = 0.040) and stimulation layers (Fig. 7*C*; *F*_(1,101)_ = 5.301, *p* = 0.023). EPSPs of PV interneurons residing in L2/3 had significantly broader half-widths in FXR1 cKO (6.07 ± 0.195 ms) than those in WT (5.22 ± 0.148 ms, *p* = 0.002). These half-widths were also significantly broader than in L4 of both WT (4.82 ± 0.138 ms, *p* < 0.001) and cKO (4.96 ± 0.194 ms, *p* < 0.001). The parallels between these results and EPSP decay-times (Fig. 6*B*) presumably reflect the contribution of decay-time to half-width (Fig. 2). Stimulation in L2/3 elicited EPSPs with significantly broader half-widths in cKO slices (5.76 ± 0.231 ms) compared with WT (4.93 ± 0.130 ms, *p* = 0.002), and this likely reflects the contribution of rise-time (Fig. 5*C*) to half-width. Thus, PV interneuron EPSP half-widths were longer in FXR1 cKO than in WT, with a greater impact of genotype on PV interneurons residing in L2/3, as well as on EPSPs elicited by stimulation of L2/3. The parallels between effects on half-width, decay-time, and rise-time provide a useful check for consistency.

## Discussion

**Figure 8.**
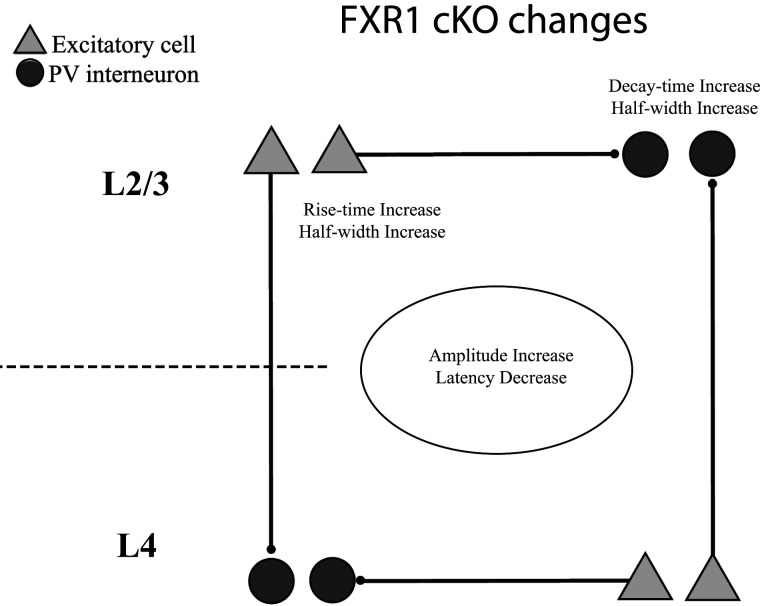
The impact of FXR1 deletion from PV interneurons on their EPSPs. Global impacts are stated within the ellipse in the center. Effects specific to residence layer are above the dark circles representing PV interneurons; effects specific to stimulation layer are below the light gray triangles representing excitatory neurons.

Experiments presented here showed that deletion of the RNA-binding protein FXR1 selectively from PV interneurons alters their responses to excitatory synaptic inputs. We used the genetically encoded hVOS to target the same PV interneurons from which FXR1 was ablated. Voltage imaging in the BC in slices of mouse primary somatosensory cortex revealed EPSPs from many neurons simultaneously across layers and with different sites of stimulation. We found that some of the effects of FXR1 cKO were global: EPSP amplitudes were larger and distance-normalized latencies were shorter. These changes were seen across layers and stimulation sites. FXR1 cKO also had selective effects, altering EPSP rise-time, decay-time, and half-width differently depending on whether PV interneurons reside in L2/3 or L4, and on which of these layers was stimulated. In general, genotype had a greater impact on PV interneurons residing in L2/3 and on EPSPs evoked by stimulating L2/3. These results are summarized schematically in Figure 8, with global changes in the center, and the residence and stimulation layer-specific changes indicated for L2/3.

Proteins in the family that includes FXR1 have both pre- and postsynaptic functions (Contractor et al., 2015). Since we measured EPSPs in the cells from which FXR1 was deleted, we first consider actions at the postsynaptic locus. PV interneuron-specific FXR1 cKO has been shown to decrease dendritic length and complexity (Shen et al., 2021). The reduced EPSP latency and larger amplitude reported here both could reflect a shorter dendritic distance from the synapse to the soma and thus less electrotonic attenuation. This could be linked to miR-124, which is expressed in PV interneurons (Abernathy and Yoo, 2015), is decreased in embryonic mouse brains lacking FXR1 (X. L. Xu et al., 2011), and can impact neuronal morphology (Chen et al., 2022). The increase in amplitude of evoked EPSCs in cortical neurons resulting from miR-124 haploinsufficiency (Kozuka et al., 2019) may be related to the increase in EPSP amplitude reported here in FXR1 cKO PV interneurons.

In WT PV interneurons, we reported that their EPSPs differ between layers of residence and layers of stimulation (Scheuer et al., 2024). Here we found that FXR1 cKO effects on rise-time, decay-time, and half-width also depended on these factors. These differences likely reflect diversity within the PV interneuron population. Basket and chandelier cells, two morphologically distinct PV interneurons subtypes, are involved in different microcircuits and have different electrophysiological properties and distributions (Povysheva et al., 2013; Q. Li and Huntsman, 2014; Almasi et al., 2019; Staiger and Petersen, 2021). The differences we observed could reflect either these well-defined morphological cell types, or finer distinctions based on gene expression profiles (Tasic et al., 2018; Gouwens et al., 2020), including different FXR1 splice variants (Mendez-Albelo et al., 2024). FXR1 expression levels may also vary between PV interneuron subtypes, and experiments in mice with heterozygous ablation may reveal graded effects. Residence layer differences would reflect the properties of PV interneuron subtypes in that layer, and stimulation layer differences would reflect the subtype targeting specificity of excitatory cell axons.

The EPSPs studied here are mediated primarily by AMPA receptors. The receptor subunit composition and the presence of auxiliary subunits have a direct impact on rise-time and decay-time (Geiger et al., 1995; Stincic and Frerking, 2015; Jacobi and von Engelhardt, 2021). Like FMR1 (Hwang et al., 2022), FXR1 controls AMPA receptor translation and trafficking (Cook et al., 2014; Khlghatyan et al., 2018a, 2020) and could thus contribute to the various changes in EPSPs reported here by altering AMPA receptor expression levels and subunit composition. While most PV interneurons do not express the GluA2 subunit, which is associated with calcium-impermeable channels and longer decay-times, Kondo et al. (1997) reported GluA2 expression in some PV interneurons in rat somatosensory cortex. Thus, FXR1 binding to GluA2 mRNA and repression of its translation (Cook et al., 2014) may be relevant to the present results. An effect on rise-time could also reflect changes in the calcium-permeable subunit GluA4, which is strongly expressed in PV interneurons and controls EPSP rise-time (Yang et al., 2011; Yamasaki et al., 2016). Additionally, PV interneuron-specific FXR1 cKO reduced expression of the T-type calcium channel Cav3.2 (Shen et al., 2021), and this could influence the shape of an EPSP. FXR1 cKO also increased the half-widths of action potentials of PV interneurons in the prefrontal cortex (Shen et al., 2021), suggesting parallel shifts of both voltage-gated channels and synaptic receptors toward slower kinetics.

The interpretations just summarized consider how the observed changes in EPSPs could arise directly from FXR1 deletion at the postsynaptic locus. However, a depressed c-FOS expression suggests that FXR1 deletion from PV interneurons reduces their electrical activity in vivo (Shen et al., 2021). This, along with the reduction in spike frequency and weaker correlation and coherence in EEG recordings, suggest an overall weakening of inhibition in the brains of these animals (Shen et al., 2021). If the primary consequence of FXR1 deletion from PV interneurons is a reduction in their output, then the increases in EPSP amplitude, shortening of latency, and increase in durations all could reflect homeostatic/compensatory responses of the network to reduced inhibition. Since the present study focused on excitatory inputs, it will be important for future studies to focus on the responses of neurons targeted by FXR1 cKO PV interneurons in order to assess the changes in their inhibitory output.

Regardless of whether the effects described here are cell autonomous or network dependent, FXR1 deletion from PV interneurons alters their EPSPs in mouse BC. The layer-specific and input-specific effects on rise-time, decay-time, and half-width reveal functional heterogeneity among the broad class of interneurons defined by the expression of PV and highlight the need for evaluating function at the level of cell type, cortical layer, and synaptic input. PV interneurons are activated rapidly by sensory inputs to provide feedforward and feedback inhibition. PV interneurons restrict temporal summation in excitatory neurons, control intracortical and thalamic integration, and participate in the generation of network oscillations (Hu et al., 2014; Ferguson and Gao, 2018). Furthermore, these roles vary between layers (Staiger and Petersen, 2021). The larger amplitude and shorter latency of EPSPs in PV interneurons lacking FXR1 have the potential to amplify their excitation and enhance their inhibitory output. This in turn could narrow the integration window in their targets, increase the sparsity of coding, and focus sensory responses. Changes in L4 are more likely to impact initial sensory responses and feedforward inhibition, whereas changes in L2/3 are more likely to impact feedback inhibition, integration, and processing.

The changes reported here in EPSP amplitude and kinetics were generally quantitative rather than qualitative. Such changes are not expected to abolish functions but have the potential to fine-tune cortical processing and alter somatosensory perception in subtle ways. Indeed, the mice studied here do not suffer gross impairment, and the subtle changes in their behavior (Shen et al., 2021) are consistent with modifications rather than impairment of circuit function. Changes in the excitation of PV interneurons could tip the balance in winner-take-all computations and shift the channeling of inter- versus intralaminar signaling. EPSPs may be impacted to lesser degrees by alterations in FXR1 expression levels or in different ways by FXR1 mutations. PV interneurons have been implicated in psychiatric disorders (Gonzalez-Burgos et al., 2015; Ferguson and Gao, 2018; Lauber et al., 2018; Nahar et al., 2021), and similar effects of FXR1 on PV interneuron EPSPs in other parts of the cortex could be relevant to the effects of FXR1 cKO on behavior (Shen et al., 2021) as well as FXR1-linked psychiatric conditions (Schizophrenia Working Group of the Psychiatric Genomics, 2014; Shen et al., 2021). It is unlikely that schizophrenia originates entirely from deficits in a single cell type such as PV interneurons, and other cell types are likely to contribute. Furthermore, the impact of FXR1 dysfunction in PV interneurons may be different when other types of neurons have deficits. Targeted deletion of FXR1 from excitatory neurons also enhances EPSPs (Cook et al., 2014). Experiments in other cell types have the potential to reveal the generality of this action and may uncover additional roles. Extending our approach to different FXR1 deficits and different types of cells will expand our understanding of the regulatory functions of FXR1 and the connection with mental illness.

## Data Availability

Data and code relevant to this study are available from GitHub at https://github.com/ksscheuer/Inter_intra_laminar and Zenodo at https://doi.org/10.5281/zenodo.11106962.

## Synthesis

Reviewing Editor: Nathalie Ginovart, University of Geneva Decisions are customarily a result of the Reviewing Editor and the peer reviewers coming together and discussing their recommendations until a consensus is reached. When revisions are invited, a fact-based synthesis statement explaining their decision and outlining what is needed to prepare a revision will be listed below. The following reviewer(s) agreed to reveal their identity: NONE.

Both reviewers expressed interest in your manuscript and agreed that it provides new insights into how FXR1 contributes to the regulation of PV interneuron activity. A major concern is the validity of defining "neurons" based on activity thresholds, as this approach likely overestimates sample sizes and impacts statistics and conclusions. Furthermore, the relevance of targeted deletion of FXR1 in PV interneurons in otherwise intact circuits to model FXR1-related psychiatric disorders should be discussed.

Please find the specific comments and queries raised by the reviewers below:

Reviewer 1:

This is an interesting dataset presenting impact of FXR1 inactivation in PV interneurons of the prefrontal cortex. This is potentially important as PV interneurons are thought to play a role in schizophrenia and that SNPs in the FXR1 gene have been associated to schizophrenia risk (GWAS) and schizophrenia symptom severity (candidate gene study).

Strengths:

Findings are not spectacular or qualitatively evident. As noted by the authors this is very credible as changes caused by schizophrenia risk genes should be subtle. We are looking into a system that remains somewhat functional and massive loss of functions would be a big surprise here.

It is also very interesting that authors found differences between interneurons of different sub-cortical layers.

Weaknesses:

The experimental system is limited by intrinsic issues.

1- The study is limited to full knockouts, Would a 50% reduction of FXR1 expression have a similar effect. This could be important as changes of FXR1 expression in schizophrenia should not result in a full loss of function.

2-FXR1 has been reported to affect excitatory synapse not only in PV interneurons but also in various types of principal neurons. These effects involved both regulation of GluA1 and GluA2 synthesis and/or trafficking. In a diseased brain one would assume that FXR1 expression should not be affected only in PV interneurons but also in principal neurons and other populations of cortical interneurons. This global effect on FXR1 expression should have consequences for network activity. The current study does not address this problem. This makes it difficult to evaluate if intrinsic effects on PV interneurons in the study are representative of what would occur in schizophrenia.

3- The study is overall descriptive as no mechanism is presented to explain changes.

Minor comments:

-It is stated that experimental animals were matched for age and sex. This is very nice but is there differences of age and sex between experimental groups used for different recording sessions? What was the sex ratio in experimental cohort?

Statistics: Authors may want to move analysis of variance and sex from the method section to the result section.

Reviewer 2:

Scheuer and colleagues examine how conditionally knocking out Fragile X autosomal homolog (FXR1) impacts the electrophysiological properties of parvalbumin interneurons in somatosensory cortex mouse brain slices. Measurements are carried out using the genetically encoded voltage indicator hVos expressed in parvalbumin neurons that are missing FXR1. The authors examine how this manipulation impacts EPSP responses in different cortical layers in response to electrical stimulation in different layers -examining amplitude and timecourse changes.

The authors show that electrical stimulation evokes voltage signals throughout the slice (Fig. 1), and measure changes in EPSP amplitude and latency in PV neurons that are missing FXR1 (and wildtype). They report differences in EPSP amplitudes and latency when pooling regions of interest within layers 3 and 4 - although the effect was not significant across layers.

Amplitude and latency did not change across layers, although the temporal properties (rise time) changed. A subset of parameters exhibited some layer-specific changes (rise times differed based on whether layer 3 versus 4 were stimulated, but not amplitude).

To advance GEVIs, it is important to begin to address important biological questions using them -- and this manuscript is an example of that. However, the effects are small and depend on (very) large sample sizes that were generated based on a statistical analysis to define clusters of pixels as neurons. Moreover, the signal-to-noise is very low which makes it difficult to believe this sensor can detect such small and subtle effects. The conclusions are also unreasonably strong for such subtle effects that may be due to sampling error. At the very least, the authors should tone down their conclusions, and reconsider whether it's appropriate to define the regions of interest they've selected as neurons.

Major.

There are well-known challenges associated with linking wide-field voltage imaging with clearly defined regions of interest. (This has driven the development of targeting motifs that restrict a GEVI to say the soma or just a handful of cells). The author's approach to define individual "neurons" based on activity above a threshold is not believable. I believe there are optical changes associated with the slice stimulation, but not that the changes are single neurons. The regions of interest in Figure 1 seem almost randomly drawn (e.g., the most strongly activated pixels don't have any regions of interest). The authors justify this approach based on a previous publication in which they show using a similar experiment that there is no correlation of amplitude or half-width with distance for different regions of interest. I imagine that a simple analysis of regions of interest that are located very close together would reveal a very strong correlation in the timecourse and amplitude of these data (which is partly supported by many similarities in the Fig. 1 traces).

The main problem with this approach is because it may be inflating the sample sizes in the data set -- the reported number of recorded neurons (1084 and 1116 in the two groups, respectively). This possibility is strongly supported by Figure 3 -- since the results are not significant when breaking the analysis apart by layer (which is reducing their statistical power).

The most harmonious solution is to avoid describing these signals as originating from individual neurons. Then the authors could report results based entirely on statistically defined regions of interest of whatever arbitrary shape they prefer. I suspect that if the authors choose a criteria that results in fewer regions of interest -- their effect will disappear. At the very least, the authors should perform a data sampling analysis to lower their statistical power to demonstrate the effects do not require such large sample sizes.

The conclusions that the authors show that FXR1 impacts varied between cortical layer and synaptic input is overstated given the signal-to-noise. A fairer conclusion may be that stimulation of mouse brain slices lacking FXR1 in PV interneurons evokes non-uniform voltage changes across a slice.

Minor.

Line 173: "or" should be "of"

Methods, data processing: The authors write that "pixels in clusters with an average SNR below a predetermined cutoff or 5 were excluded". Does this mean that within a single region of interest, single pixels could be excluded if they did not meet the statistical criteria? If so I think this is a questionable way to define a region of interest.

Methods: The authors describe a data set under "data processing and analysis" (WT 7 animals/38 slices; cKO 7 animals/33 slices). It seems like the same data set is again described under "experimental design and statistical tests".

## Author Response

Dear Dr. Ginovart, Thank you for inviting us to submit a revised version of our manuscript. We appreciate the positive comments and constructive criticism, and have made every effort to address all of the reviewers' concerns. Our revised manuscript highlights new and edited text.

Synthesis Statement for Author (Required):

Both reviewers expressed interest in your manuscript and agreed that it provides new insights into how FXR1 contributes to the regulation of PV interneuron activity. A major concern is the validity of defining "neurons" based on activity thresholds, as this approach likely overestimates sample sizes and impacts statistics and conclusions. Furthermore, the relevance of targeted deletion of FXR1 in PV interneurons in otherwise intact circuits to model FXR1-related psychiatric disorders should be discussed.

With regard to defining neurons, we expanded our summary of our recently published method (Scheuer et al. 2024) and added detail to address these concerns (P. 6-7). This makes clear that our criteria for cell selection was extremely conservative and does not oversample. We also added a qualification (P 7, lines 197-199) and moderated the language.

We expanded our discussion of relevance, and added qualifications about roles of FXR1 in other cell types (P. 18-19, lines 485-488 and 491-495). There is a considerable body of research implicating PV interneurons in mental illness (Gonzales-Burgos et al, 2015; Lauber et al., 2018; Nahar et al., 2021). Especially relevant is a report from our collaborator's lab showing deficits in the same genetic model we used in our study (Shen et al., 2021, FXR1 regulation of parvalbumin interneurons in the prefrontal cortex is critical for schizophrenia-like behaviors. Mol Psychiatry 26:6845-6867).

Details are presented below in response to the specific criticisms.

Reviewer 1:

This is an interesting dataset presenting impact of FXR1 inactivation in PV interneurons of the prefrontal cortex. This is potentially important as PV interneurons are thought to play a role in schizophrenia and that SNPs in the FXR1 gene have been associated to schizophrenia risk (GWAS) and schizophrenia symptom severity (candidate gene study).

Strengths:

Findings are not spectacular or qualitatively evident. As noted by the authors this is very credible as changes caused by schizophrenia risk genes should be subtle. We are looking into a system that remains somewhat functional and massive loss of functions would be a big surprise here.

It is also very interesting that authors found differences between interneurons of different sub-cortical layers.

Weaknesses:

The experimental system is limited by intrinsic issues.

1- The study is limited to full knockouts, Would a 50% reduction of FXR1 expression have a similar effect. This could be important as changes of FXR1 expression in schizophrenia should not result in a full loss of function.

Gene dose is an important consideration. It is now addressed on P 17, lines 432-434 as well as in our discussion of schizophrenia on P 18, lines 485-486.

2-FXR1 has been reported to affect excitatory synapse not only in PV interneurons but also in various types of principal neurons. These effects involved both regulation of GluA1 and GluA2 synthesis and/or trafficking. In a diseased brain one would assume that FXR1 expression should not be affected only in PV interneurons but also in principal neurons and other populations of cortical interneurons. This global effect on FXR1 expression should have consequences for network activity. The current study does not address this problem. This makes it difficult to evaluate if intrinsic effects on PV interneurons in the study are representative of what would occur in schizophrenia.

This is an important point that a mutation that alters mental health is be global. We now note the roles of FXR1 in other cell types, and address the distinction between mutants and knock-outs (P 18-19, lines 491-495). The schizophrenia-like behaviors resulting from targeted deletion of FXR1 from PV interneurons (Shen et al., 2021) strengthens the relevance.

3- The study is overall descriptive as no mechanism is presented to explain changes.

We had discussed potential mechanisms in terms of glutamate receptors, calcium channels and micro RNA (P 16, lines 419-421 and P 17, lines 437-453). Without doing more experiments we cannot elaborate beyond what we already said. If there is a specific point we failed to mention we would be happy to add it.

Minor comments:

-It is stated that experimental animals were matched for age and sex. This is very nice but is there differences of age and sex between experimental groups used for different recording sessions? What was the sex ratio in experimental cohort? The ratio was added to P 9, line 239-240.

Statistics: Authors may want to move analysis of variance and sex from the method section to the result section.

We believe presenting statistical methods in the Methods section follows journal guidelines.

Reviewer 2:

Scheuer and colleagues examine how conditionally knocking out Fragile X autosomal homolog (FXR1) impacts the electrophysiological properties of parvalbumin interneurons in somatosensory cortex mouse brain slices. Measurements are carried out using the genetically encoded voltage indicator hVos expressed in parvalbumin neurons that are missing FXR1. The authors examine how this manipulation impacts EPSP responses in different cortical layers in response to electrical stimulation in different layers -examining amplitude and timecourse changes.

The authors show that electrical stimulation evokes voltage signals throughout the slice (Fig. 1), and measure changes in EPSP amplitude and latency in PV neurons that are missing FXR1 (and wildtype). They report differences in EPSP amplitudes and latency when pooling regions of interest within layers 3 and 4 - although the effect was not significant across layers.

Amplitude and latency did not change across layers, although the temporal properties (rise time) changed. A subset of parameters exhibited some layer-specific changes (rise times differed based on whether layer 3 versus 4 were stimulated, but not amplitude).

To advance GEVIs, it is important to begin to address important biological questions using them -- and this manuscript is an example of that. However, the effects are small and depend on (very) large sample sizes that were generated based on a statistical analysis to define clusters of pixels as neurons. Moreover, the signal-to-noise is very low which makes it difficult to believe this sensor can detect such small and subtle effects. The conclusions are also unreasonably strong for such subtle effects that may be due to sampling error. At the very least, the authors should tone down their conclusions, and reconsider whether it's appropriate to define the regions of interest they've selected as neurons.

The signal-to-noise ranged from 5.06-26.25 for all cells included in our analysis (P. 7, line 197). By sampling error we assume the reviewer means that our criteria for identifying neurons introduces a bias between wild type and cKO. We imposed the condition of using only data where we were able to identify 8 or more neurons in a layer (P 9, lines 242-244). This avoided a form of sampling error in which results may be skewed by slices/layers with only a few resolvable neurons. Our addition of important details to the Methods section (described below) address additional concerns.

Major.

There are well-known challenges associated with linking wide-field voltage imaging with clearly defined regions of interest. (This has driven the development of targeting motifs that restrict a GEVI to say the soma or just a handful of cells). The author's approach to define individual "neurons" based on activity above a threshold is not believable. I believe there are optical changes associated with the slice stimulation, but not that the changes are single neurons. The regions of interest in Figure 1 seem almost randomly drawn (e.g., the most strongly activated pixels don't have any regions of interest). The authors justify this approach based on a previous publication in which they show using a similar experiment that there is no correlation of amplitude or half-width with distance for different regions of interest. I imagine that a simple analysis of regions of interest that are located very close together would reveal a very strong correlation in the timecourse and amplitude of these data (which is partly supported by many similarities in the Fig. 1 traces).

Our selection of regions of interest was based on much more than an intensity threshold. Our recent publication (Scheuer et al., 2024) provided a very detailed account of this method and Fig. 2 of that paper presents a flowchart with 11 steps. We actually employed a joint threshold, identifying responsive neurons in both maps of peak amplitude and maps of SNR. We used only small groups of spatially contiguous pixels with SNR in the same k-means level. A paragraph from lines 162-199 has been greatly expanded and edited to make these points and clarify the process.

The reviewer is concerned that regions in Fig. 1 with strongly activated pixels do not have any cells outlined. This was intentional. These regions are large and must contain many cells crowded together (now pointed out on P 7, lines 206-208). It is impossible to find single cells in such regions. We had stated that regions larger than a single cell were excluded (P 6, line 171) and added more detail (P 6-7, lines 171-178). We now state the mean pixel numbers {plus minus} SD of accepted regions (P 6, line 172). Their small sizes (average < 3 pixels) and uniform SNR (by k-means clustering) further supports their assignment as single neurons. Each accepted region of interest is spatially distinct. Nevertheless, a region could contain more than one neuron and we have added this qualification (P 7, lines 197-199) The reviewer is skeptical about our validation based on lack of correlation in plots of amplitude and half-width versus distance, asking if there is a greater correlation when regions of interest are close together. As we now point out (P 7, lines 176-178), regions or interest are excluded if they have any contact with another region of interest, even at a single vertex. Since the significance of our previously reported lack of correlation in amplitude-distance plots and half-width-distance plots may not be clear, we added a sentence about the interpretation of this result, and refer to reports showing that when regions contain multiple cells these plots showed decreasing amplitude and increasing half-width with distance (P 7, lines 186-188).

The main problem with this approach is because it may be inflating the sample sizes in the data set -- the reported number of recorded neurons (1084 and 1116 in the two groups, respectively). This possibility is strongly supported by Figure 3 -- since the results are not significant when breaking the analysis apart by layer (which is reducing their statistical power).

The most harmonious solution is to avoid describing these signals as originating from individual neurons. Then the authors could report results based entirely on statistically defined regions of interest of whatever arbitrary shape they prefer. I suspect that if the authors choose a criteria that results in fewer regions of interest -- their effect will disappear. At the very least, the authors should perform a data sampling analysis to lower their statistical power to demonstrate the effects do not require such large sample sizes.

Our criteria for selecting regions of interest were extremely conservative, and excluded ambiguous cases. Because we had the same concern expressed by the reviewer, we implemented the following. "For a given slice, parameters from PV interneurons in layers with 8 or more responsive cells were averaged for each layer, and that layer average was taken as the unit of analysis for statistical testing (n = 55 WT, n = 54 cKO)" (P. 9, lines 242-244). In response to the reviewer's concern we added a qualification on P 7, lines 197-199, and used more cautious wording (P. 7, lines 182-183).

The conclusions that the authors show that FXR1 impacts varied between cortical layer and synaptic input is overstated given the signal-to-noise. A fairer conclusion may be that stimulation of mouse brain slices lacking FXR1 in PV interneurons evokes non-uniform voltage changes across a slice.

We hope that the above responses to the concerns about selection of regions of interest strengthen this conclusion. Stating that FXR1 ablation has a non-uniform impact immediately raises the question of how impacts vary spatially. This would leave the issue hanging and create a large void.

Minor.

Line 173: "or" should be "of" Corrected Methods, data processing: The authors write that "pixels in clusters with an average SNR below a predetermined cutoff or 5 were excluded". Does this mean that within a single region of interest, single pixels could be excluded if they did not meet the statistical criteria? If so I think this is a questionable way to define a region of interest.

This refers to k-means defined clusters not to regions of interest. We have clarified our exclusion criteria (P 7, lines 178-180).

Methods: The authors describe a data set under "data processing and analysis" (WT 7 animals/38 slices; cKO 7 animals/33 slices). It seems like the same data set is again described under "experimental design and statistical tests".

The redundant text has been removed (P 7, lines 195-196).

We thank the reviewers and editor for their criticism and hope the paper is now acceptable.

Sincerely Meyer Jackson
